# Loneliness and Intersectional Discrimination Among Aging LGBT People in Spain: A Qualitative Research Study of Gay Men

**DOI:** 10.3390/bs15070846

**Published:** 2025-06-23

**Authors:** Sacramento Pinazo-Hernandis, Jose Miguel Cerezo, Celia Carrascosa

**Affiliations:** Department of Social Psychology, University of Valencia, 46010 Valencia, Spain; jomarce@alumni.uv.es (J.M.C.); celia.carrascosa@uv.es (C.C.)

**Keywords:** loneliness, gay men, men sex men, discrimination, homosexuality, aging

## Abstract

Background: Loneliness is both a social and a health-related problem, and among LGBT people, feelings of loneliness are often exacerbated. According to the minority stress theory, stress and loneliness can be directly related to the discrimination and stigma that are experienced over time in a heteronormative society. Exposure to social stigma causes mental health problems, stress, and loneliness, coupled with increased social and economic vulnerability. Method: The aim of this study was to conduct an exploratory analysis of homosexual men’s feelings of loneliness and their relationship with experiences of discrimination throughout their life. A qualitative methodology involving focus groups and individual interviews was utilized. Results: The results show the double or overlapping discriminations that gay men experience as a result of their age and sexual orientation and, in some cases, their HIV status, and the relationship between said discrimination and the feeling of unwanted loneliness. Exposure to such intolerance is more common among those with a reduced social network, which limits their social participation and has a negative impact on their well-being. Conclusion. The aging process of LGBT people implies a new context of intervention and research that must be addressed to prevent episodes of unwanted loneliness that negatively affect the quality of life among this demographic.

## 1. Introduction

Loneliness has been widely recognized as a critical public health issue on a global scale. Despite the fact that a substantial body of research has been dedicated to examining the prevalence and impact of loneliness among the elderly population in Spain, there remains a conspicuous absence of empirical evidence addressing the specific experiences of loneliness among older individuals from ethnic minority backgrounds and those identifying as lesbian, gay, bisexual, or transgender (LGBT).

Loneliness has been defined as an emotional state of dissatisfaction that people experience, and can be categorized as either situational, defined as an episodic experience, or chronic, characterized by protracted feelings of loneliness (Hermann et al., 2022; Gorczynski & Fasoli, 2021; Peplau & Perlman, 1982). Regardless of its duration, loneliness is an important social and health issue that many people face throughout their lives. Risk factors are of paramount importance, encompassing cultural, social, interpersonal, and individual factors.

Although a person may feel lonely at different points in their life, some studies, such as that of Pinquart and Sorensen (2001), have identified a correlation between increasing age and heightened feelings of loneliness. However, the relationship remains inconclusive, as not all research consistently supports this link. In studies that do report greater loneliness among older adults, this trend is often attributed to factors such as reduced social interaction, the loss of close relationships (e.g., a spouse or friends), declining health, or diminished mobility, all of which may limit opportunities for social engagement.

### 1.1. The Experience of Loneliness Among LGBT Older Adults During the Pandemic

It has become increasingly clear that the COVID-19 pandemic has had a negative impact on socially or economically disadvantaged people, as well as older adults, as it interacts with and exacerbates existing health or social inequalities (McGowan et al., 2021). So far, there are few available data on the impact of pandemic-related confinement and other restrictions on people from minority communities, such as marginalized genders (Armitage & Nellums, 2020; Barreto et al., 2025). Along these lines, a United Nations (UN) report involving more than 100 countries highlights the negative implications of the pandemic for LGBT people (UN, 2020), a finding corroborated by subsequent studies, such as the study by Nowaskie and Roesler (2022).

In their study on the impact of COVID-19 confinement on LGBT adults with disabilities, Platero and López-Sáez (2021) found that some of the specific risks faced by this population were increased control due to intensive cohabitation with family members and having their social contacts restricted, which established concrete barriers to their sexual rights; fear of discrimination was also reported during the pandemic, due to news coverage about harassment against LGBT people during this period. Meanwhile, in their studies, Akré et al. (2021), Armitage and Nellums (2020), and Bavinton et al. (2022) highlighted the broad impacts on this population, as well as on health care and other frontline workers, due to high levels of stress and the risks of exposure to COVID-19 during the pandemic. In light of this, research on health and health-related behaviors is important to improve our understanding of these issues.

### 1.2. LGBT Ageing, Loneliness and Social Support

During the aging process, a person’s main source of support tends to be their nuclear family. However, many older people especially within the LGBT community have not had children, and/or have no relationship with their family of origin. In these cases, any support that is needed later in life is unlikely to be provided by their family, and they may suffer from greater loneliness as a result (Ribeiro-Gonçalves et al., 2021).

Considering this dissociation from the birth family, Weston’s (1991) seminal study examined how the families of gay individuals defy the traditional adage that “one can choose one’s friends, but not one’s family”, thereby giving rise to the concept of a chosen family. Such families may not consist exclusively of friends; they may also include lovers, adopted children, children from previous heterosexual marriages, and children fathered by artificial insemination. Although the discourse on the gay family features familial symbols, such as blood, choice, and love, it redirects those symbols toward the task of demarcating a different category of family.

The empirical research confirms the unique vulnerabilities faced by LGBT older adults. For example, Yang et al. (2018) found that LGBT older adults are more likely to live alone and less likely to have children than their heterosexual and cisgender counterparts. This absence of a close family network increases the risk of social isolation and its associated consequences. Fokkema and Kuyper (2009) found that homosexual men, in particular, were significantly more likely than heterosexual men to have lost a partner and to live alone. Among women, the opposite trend was observed. Consequently, LGBT older adults—especially men—are significantly more likely to experience feelings of loneliness.

There is strong evidence linking loneliness among LGBT older adults to poor mental health outcomes. Shrira et al. (2020) and Wittgens et al. (2022) reported a correlation between loneliness and mental health issues, including self-harm and suicide attempts. In the United Kingdom, McCann and Brown (2019) found that LGBT individuals face a 1.5 times greater risk of depression, anxiety disorders, alcohol abuse, and suicidal ideation compared to the general population.

The study by King et al. (2020) further highlighted this disparity, noting a significant increase in the lifetime prevalence of suicide among gay and bisexual men compared to the general population. These findings align with those of Kim and Fredriksen-Goldsen (2014), who demonstrated that gay and bisexual men were more likely to experience loneliness than lesbian and bisexual women.

Finally, Lin et al. (2024) explored the mediating role of loneliness in the relationship between perceived and internalized sexual stigma and suicide among gay and bisexual men. Their results indicate a strong positive association, particularly among older participants, underscoring the compounded effects of ageing, stigma, and social isolation on mental health within this population.

### 1.3. Discrimination, Stigma and Mental Health in LGBT People

Goffman (1963) defines stigma as a condition, attribute, trait, or behavior that causes the carrier person to be included in a social category whose members are perceived as unacceptable or inferior. Stigma is conceptualized as the processes of labeling, stereotyping, separating, and discriminating individuals or groups based on distinguishing characteristics, such as physical disabilities and mental disorders (Link & Phelan, 2001). Conditions in the social environment, in addition to personal events, are sources of stress that can have an effect on people’s mental health. People who belong to stigmatized social categories, such as the LGBT community, are significantly impacted by social stress. Homophobia-related prejudice and discrimination can be stressful. Self-perceived stigma (or internalized homophobia)—a process in which marginalized individuals become aware of public stigma, agree with others’ stereotypical views of them, and eventually internalize these views by applying them to themselves—are also cause a concern. The acceptance and internalization of such derogatory and undermining views can lead to low self-esteem, loneliness, and self-isolation (Cohn-Schwartz et al., 2025).

Lim et al. (2021) discussed the impact of stigma and discrimination and how prejudice targeted towards gay men in Asia may have undesirable physical, psychological, and social consequences. According to their findings, 32% of gay older people in their study had experienced verbal harassment, 23% had encountered discrimination, and 8% had been physically assaulted, all in the previous 12 months (Balaji et al., 2017). These findings were confirmed by authors such as Perone et al. (2020), who stated that LGBT older adults face greater risks of social isolation due to the decades of discrimination they have experienced, and this isolation becomes an additional factor for vulnerability to loneliness. Only when sexuality is understood in terms of social and historical analysis will a more realistic sexual policy be possible.

In 1952, the first edition of the Diagnostic and Statistical Manual of Mental Disorders (DSM) was published by the American Psychiatric Association. In that initial version, homosexuality was classified as a symptom of mental illness, based on unsubstantiated theories that linked same-sex attraction to psychological disorders. This classification contributed to significant controversy and criticism, prompting successive revisions of both the DSM and the International Classification of Diseases (ICD) over time. Ultimately, in 1980, the DSM-III removed homosexuality as a diagnostic category (Peidro, 2021).

Making homosexuality a diagnosable condition reinforces the idea that there is a “healthy” sexuality in contrast to a “sick” one. When legitimized by scientific disciplines, such as psychiatry and psychology, a diagnosis not only defines a disorder but implies the need for treatment, thereby perpetuating marginalization. Many individuals who came of age during that era were forced to conceal their sexual identity, living double lives marked by fear, chronic stress, and internalized stigma—spending more of their lives under the label of “ill” than with an affirmed sexual identity.

Despite the removal of homosexuality from diagnostic manuals, this form of discrimination persists in various forms today.

According to the most recent data published by the Spanish Ministry of the Interior, hate crimes in Spain rose by 21.3% in 2023 compared to the previous year, with a total of 2,268 recorded incidents. Of these, 522 offenses were related to sexual orientation and gender identity, constituting approximately 23% of all reported hate crimes. This positions such offenses as the second most prevalent category, surpassed only by those motivated by racism and xenophobia, which accounted for 856 cases (41.8%).

These figures mark a notable increase from the previous years. For instance, the 2021 Hate Crime Survey from the same Ministry revealed that 35.47% of the 437 surveyed cases cited sexual orientation or gender identity as the principal motive for discrimination. The 2023 data thus reflect not only an escalation in the absolute number of reported cases but the persistent vulnerability of LGBT individuals to hate-motivated violence and discrimination.

This upward trend underscores the urgent need for more robust public policies and protective measures to safeguard the rights and wellbeing of LGBT populations in Spain. Although significant progress has been made in terms of formal legal recognition of LGBT rights, beginning with the protests of the 1970s and culminating in the establishment of regional laws, such as Law 14/2012 of the Basque Country on gender identity and trans rights, social equality remains unevenly realized. While most Autonomous Communities now have legislation promoting equality and non-discrimination, these legal frameworks are still relatively recent and their implementation varies.

The empirical evidence continues to reveal high levels of discrimination. A national study by Elipe et al. (2020) found that 66% of homosexual individuals in Spain had experienced insults or disrespect based on their sexual orientation, 71% reported having been mocked, and 35% had received threats. More recent data from the 2025 “Estado del Odio/State of Hate” report by the Federación Estatal LGTBI+ has shown that one in five LGBT+ individuals has experienced harassment within the past year, and 42.5% have encountered hate-related situations. Incidents of physical or verbal aggression rose from 6.8% to 16.25%, affecting an estimated 812,000 individuals. These aggressions primarily occurred in public spaces, followed by educational settings, leisure environments, family contexts, and workplaces.

Furthermore, a 2024 report by the European Union indicated that 53% of LGBT+ individuals in Spain had experienced some form of harassment in the preceding year highlighting the structural and persistent nature of this issue across national contexts.

Collectively, these findings emphasize the enduring challenges faced by sexual and gender minorities in Spain. They also reinforce the imperative for sustained policy intervention, public awareness, and the strengthening of institutional responses to ensure equality, safety, and dignity for LGBT communities.

Such discriminatory experiences frequently lead to heightened feelings of loneliness, diminished social support networks, increased stress, and experiences of misunderstanding and rejection, particularly within family contexts, and broader social exclusion (Fish & Weis, 2019). Authors such as Mahon et al. (2021) have observed that the prevalence of social anxiety disorder is higher in sexual minorities compared to heterosexual people due to the discrimination experienced and felt throughout their lives, which makes it difficult to establish or maintain social ties.

*Minority stress theory* (Meyer, 2003; Fulginiti et al., 2021; Grigoreva & Szaszkó, 2024; Mongelli et al., 2019) states that sexual minorities are exposed to social stigma and, throughout their lives, they face many stressful psychosocial events and stigmatizing conditions at the structural level in a heteronormative society. Such conditions can exacerbate the development of various mental health problems, stress, and loneliness, coupled with increased social and economic vulnerability.

### 1.4. Ageism or Age Discrimination

In the case of older LGBT people, in addition to possible discrimination based on their sexual orientation or gender identity, ageism is also a cause for concern. Experiences of prejudice or discrimination toward people due to their age, known as *ageism* (Nelson, 2005), have been associated with poorer well-being in general populations. Butler and Lewis defined ageism as “a process by which older people are systematically stereotyped as being old, in the same way that racism and sexism act, in which cases it is due to skin color or gender” (Butler & Lewis, 1973, p. 141). One of the ways in which an ageist society manifests itself is through the conception of older adults as a homogeneous and asexual whole.

Prejudices towards older people are also found within the LGBT community itself, where ideas about youth and old age are especially prevalent among homosexual men due to the association of homosexuality with attributes such as youth and beauty (Dellers et al., 2025).

Among gay men, experiences of ageism and sexuality acceptance concerns predict poorer outcomes on all the well-being measures. In addition, gay men who were higher on sexuality acceptance concerns had higher psychological distress and lower resilience, but only when they also had greater experiences of ageism (Lyons et al., 2022).

Research has consistently found that sexual minority men (gay and bisexual men) report disproportionately higher mental health concerns compared to heterosexual men (Argyriou et al., 2021).

Given the intersection of ageism and discrimination based on sexual orientation or gender identity, and their documented negative impact on the mental health and well-being of older LGBT individuals—particularly gay men—it becomes essential to further investigate this population’s unique experiences and needs.

This study aims to explore the experiences of loneliness among homosexual men in later life, with particular attention to how discrimination and stigma shape and contribute to these experiences throughout the aging process. Experiences of loneliness during the COVID-19 pandemic will also be taken into consideration because our research was conducted in times of confinement.

## 2. Method

### 2.1. Participants

The participants of this study were 20 gay men living in Spain, aged between 43 and 68 years (M = 56.5, SD = 7.47), representing middle-aged and older adults; 50% of the participants lived alone. Among the participants, seven were cohabiting with a partner; ten reported experiencing health conditions or disabilities; and twelve were either retired, unemployed, or engaged in part-time employment (Table 1).

Three focus groups were conducted with 5 participants each group (15 participants in total, FG1: I1 to I5; FG2: I6 to I10; FG3: I11 to I15); several sessions were held for each group, in addition to individual interviews with another 5 participants (I16 to I20). This research design was intended to support the comfort of the participants in order to facilitate the depth of information obtained. Data collection was conducted over a continuous 18-h timeframe. The inclusion criteria were as follows: identifying as a homosexual man, being over 40 years old, living in Spain, and being able to participate in online discussion groups, which implies having no sensory impairments that could hinder participation, and having access to an internet connection at home.

### 2.2. Instruments

Interviews and focus groups were used in this research. The interview allowed us to inquire about different topics of interest in an individualized manner, collecting information via open questions. Focus groups are a planned group conversation, in which several people answer questions and discuss a common topic, led by the interviewer where needed. This group interaction facilitates and enhances the relevant narratives (Krueger & Casey, 2015).

### 2.3. Procedure

The Senior Citizens Group of the Lambda Association, LGBT collective, was informed of the project and the objectives of the research, and were responsible for informing the participants about the nature of this study. The total group of this association comprised 32 individuals, of whom 15 took part in the focus group—a relatively high proportion of the overall sample, nearly 50% of the overall sample Additionally, five participants were interviewed individually. Since this research took place during the COVID-19 pandemic, the focus group and the interviews were conducted online via Zoom. The majority of the participants reported being regular internet users and demonstrated familiarity with the Zoom platform; those who were unfamiliar with it were provided with written instructions. No interviews had to be repeated. Once saturation was achieved, no more interviews were conducted. This study complies with the COREQ Guidelines for research using qualitative methodology (Tong et al., 2007).

The confidentiality of the participants’ data and personal information was assured and the ethical criteria established in the 64th General Assembly of the Declaration of Helsinki were taken into account. All the security measures outlined in the Regulations for the Development of Organic Law 3/2018 on Personal Data Protection and Guarantee of Digital Rights were adhered to. Participants were informed of the objectives of the research, the interview procedure, and how their information would be handled.

This study was conducted according to the guidelines of the Declaration of Helsinki, and approved by the University of Valencia Ethics Committee. Informed consent was obtained from all the subjects involved in this study.

The focus groups and interviews were conducted by two of the authors of this article, who have received specialized training with LGBT people and in gerontology; all interviews were recorded in audio format and transcribed manually.

The interviewers used memos that later helped with the completion of the information. To guarantee the anonymity of the interviewees, each participant was assigned an alphanumeric code (I1, I2, I3, etc.).

Based on the literature and the previous theoretical approach, an interview script was prepared, featuring topics that served as a reference for the development of the sessions. The questions were open-ended, allowing them to be more easily adapted to the needs of the exploratory research and the characteristics of the participants so that they could express their opinions freely, improving the quality of the data. The interview consisted of the following parts: social relationships, social support, loneliness, discrimination, and social isolation.

### 2.4. Analyses

The ATLAS.ti program was used for data extraction. The narratives were analyzed using a deductive–inductive procedure described by Glaser and Strauss (1967), which comprised the following steps: familiarization with the data, generation of initial codes by coding the initial ideas, a search for themes, followed by a review, definition, and selection of the testimonies that best reflect each theme. Thus, an initial floating reading was performed to gain familiarity with the texts; the texts were separated into units, which were then categorized by themes, and coded by subcategories. Next, for the data analysis, a thematic analysis was performed. We selected this method to identify, analyze, and report the patterns (themes) within the data, to organize and describe the dataset in detail, and to illustrate the frequency with which the same information was reported by different groups. The category coding process was performed independently by researchers 1 and 2 and supervised by researcher 3 until inter-rater validation was achieved.

## 3. Results

The participants talked about their loneliness and lived experiences, differentiating between different forms of loneliness within their narratives. Although the participants experienced and perceived loneliness differently, unwanted loneliness and its link with discrimination and stigma was the topic that generated the greatest interest, dialogue, and the greatest amount of repeated information, especially among the older participants who lived alone. It should also be noted that the experiences of loneliness had accompanied all of the interviewees throughout their lives. The participants spoke of loneliness and associated it with their older age, with deprivation and loss, with stigma and discrimination (twofold, for age and for belonging to the LGBT collective; or threefold, for those who had HIV), with the generational and digital gap, with the lack of meeting and socialization spaces, and with the COVID-19 pandemic. They also talked about the coping strategies they had developed to reduce the loneliness.

The data or recurrent themes were organized through open coding, with 26 codes identified overall. Then, categorization was performed to identify the semantic content patterns, resulting in 4 categories and 17 subcategories (Table 2). The discourses were approached according to the nexus of selected concepts and codes, with a total of 377 verbatim phrases obtained in the coding. The categories were informed by the following criteria: deriving from a single classification principle, homogeneity of the criteria among judges, objectivity, fidelity, relevance, pertinence, exhaustivity, independence, and mutual exclusivity.

Table 2 summarizes the different codes and showcases the results obtained.

## 4. Chosen or Desired Solitude

Loneliness is a complex experience that can sometimes be sought or desired and understood as a positive feeling. It becomes meaningful when balanced with social interactions, especially during moments when support and companionship are needed. As one participant expressed the following:

“Of course, there are times of the day when I feel like taking and being at home alone… but always knowing that I have the possibility, that if I want, I can call a friend and talk to him on the phone or meet him for a coffee or go to visit my family”.

(I11)

This highlights that the feeling of loneliness is not merely about physical solitude, but rather about the availability and quality of social connections. Indeed, living alone does not necessarily equate to feeling lonely. Some individuals who live alone report not experiencing loneliness, emphasizing the importance of social contact even when physically by themselves. As another participant stated the following:

“I live alone, I am alone for many years, but I don’t feel alone. You call someone, just as they call you, and you don’t feel lonely”.

(I13)

These reflections illustrate that loneliness is subjective and depends largely on one’s perception of social support and connection, rather than just physical presence or absence of others.

## 5. Undesired Loneliness

Drawing upon the findings from the interviews, it is evident that unwanted loneliness can stem from a multitude of factors, including but not limited to specific situations and cumulative effects. These factors encompass being part of the LGBT collective, as well as advanced age, discrimination, a paucity of social acceptance, a paucity of self-acceptance, which can lead to isolation and the absence of relationship spaces, of contact with other generations, and even of technological apparatus or knowledge in a digital society.

### 5.1. Loneliness Linked to Old Age and Loneliness Linked to LGBT Identity

Some interviewees expressed that loneliness is an implicit and inherent aspect of old age, while others explicitly linked loneliness to their experience as members of the LGBT community. The findings suggest that, for many participants, loneliness is a constant presence in the life of an LGBT person—almost like a lifelong companion, as one participant reflected as follows:

“A little theory I have is that I believe that homosexuals have a concept of loneliness that does not make us realize that it exists, that is to say, it has accompanied us since we were small, because in spite of everything, in adolescence, we have suffered from it. Some more and others less, but because of the time, the parents, the experience… Loneliness is like a traveling companion that is always there and we never realize how big it can be or even think it does not exist, when in reality I believe that loneliness is always with us, it is inherent to homosexuality”.

(I9)

Another participant highlighted the compounded effect of aging on this feeling:

“There is a certain loneliness and sadness to which is added the loneliness of old age… Because there are things that are difficult to communicate and sometimes it is a relief to be able to talk about certain issues with people”.

(I17)

Finally, the sense of inevitable solitude within the LGBT experience was expressed as follows:

“LGBT people are destined to be left alone, in a way. And, well, when you are young you are on your own and you can do everything without needing anyone, but when you are older, obviously, we always end up, all society or all people, needing some help”.

(I20)

It is evident from these reflections that loneliness is perceived not only as an inevitable consequence of the ageing process, but as a profoundly intertwined aspect of the lived experience of individuals identifying as LGBT.

### 5.2. Loneliness Due to Losses and Shortages

The loss or absence of people and the lack of affection can also lead to unwanted loneliness. Three aspects related to losses were identified, including the loss of a partner, HIV as a trigger for many losses (especially in the 1980s–1990s), and the absence of family support. Below are some quotations that exemplify this subcategory.

#### 5.2.1. Loss of Partner or Fear of Damaging Relationships

The presence of a partner is a fundamental aspect of many people’s lives, and the loss of a partner often leads to profound feelings of unwanted loneliness. As one participant shared the following:

“Loneliness is something I have always known; it is not unfamiliar to me. I have often felt alone, even when I had a partner. When those relationships ended, the sense of loneliness was especially intense. In other words, loneliness is a constant presence in my mind”.

(I12)

Additionally, some interviewees explained that their tendency to isolate themselves stemmed from a fear of being hurt, pain they had experienced previously:

“We have all been hurt in one way or another, and those wounds make you want to be alone”.

(I14)

These testimonies highlight how both the loss of intimate relationships and past emotional pain contribute significantly to feelings of loneliness and self-isolation.

#### 5.2.2. HIV Losses

The HIV/AIDS pandemic of the 1980s and 1990s in Spain took the lives of many young people, including a significant number of gay men, and these losses reduced the number of friendships or partners of many people. The associated health issues and social stigma further limited their social participation and, therefore, their relationships and social networks. One participant vividly recalled the profound losses experienced during that period:

“At the time when I was supposed to have lifelong friends, AIDS came along and took 80% of the people away from me. Well, if I knew 15 guys, out of those 15, I’m sure that 3 or 4 of them would now be my ‘lifelong friends’, hell, that’s a treasure. Then, of course, and then the death of my partner happens to me, and then the death of my brother happens to me, fuck, but shit, what is this? Then, the guy you love the most, the one who is your best friend and the one who, once you have passed the question of a partner, is no longer a strong friendship since… Fuck, the first love! In the sense that he goes and gets infected and that lasts 13 years, and that 8 months before starting to have some good medicines, because he started taking AZT, he dies. Fuck! I’m going from pill to pill, hey!”.

(I3)

He also reflected on the loss of friends in their prime:

“I’m talking about guys in their 30s and 40s, in their prime, sentenced to death at the time. Of all these friends we had, this whole group … they were disappearing, just like that, so quietly.”

(I3)

These testimonies illustrate how the HIV/AIDS crisis profoundly disrupted social bonds and left lasting scars on the LGBT community’s social fabric.

#### 5.2.3. Lack of Family Support

Family support and reciprocal assistance within families play a crucial role in individuals’ personal, familial, and social development. Such support helps people navigate crises and overcome adversity throughout their lives. Conversely, the absence of a supportive family network or the lack of healthy, functional relationships characterized by secure attachment and mutual care can contribute significantly to feelings of loneliness. Moreover, not feeling truly part of one’s family exacerbates this sense of isolation.

One participant reflected on their lifelong experience of loneliness despite living with family:

“Throughout my life I have always felt very lonely. Even when I lived with my family, I was absolutely lonely because we lacked communication; it was like a real book, and it was very big”.

(I5)

Another shared the deep loneliness stemming from early detachment from parents and the challenges of accepting their sexual identity:

“I have felt loneliness since I was very young. I suffered a lot of detachment from my parents; that loneliness has been very hard for me to assimilate. In my adolescence and early youth, I felt it even more strongly, especially when I began to have relationships and had to accept my homosexuality. That feeling of loneliness has been very deep and has lasted for many years, always”.

(I12)

A further participant described the painful exclusion experienced within family dynamics after his brother’s illness and death:

“To add to my loneliness… I had a brother two years older than me, and when he was 59, he got sick. I could see how close his wife’s family was. Then I realized, with great sorrow, that I was not really part of that family. I was ‘the brother,’ but not truly inside. Not even a phone call asking how I was. When my brother died, that family slowly dissolved for me. I have no relationship with them anymore. They said that my being homosexual didn’t matter, but when the moment of truth came, it did. I have spent years without anyone calling me at Christmas to ask how I was or if I was coming. It’s a tremendous hardship I have had to endure”.

(I3)

Family support is particularly important for LGBT individuals, yet many lack the traditional family networks that heterosexual people might have. While a minority of LGBT people have adopted children or formed families that provide some support, most are either in partnerships without children or live alone.

“Family support is important; among LGBT people, only a minority have adopted or have children who can offer support. Generally, LGBT individuals are either partnered or alone because they do not have the family network that heteronormative people often rely on”.

(I1)

This absence raises the following common concern:

“We don’t have children, so who will take care of us?”.

(I9)

### 5.3. Loneliness Due to Discrimination

One of the main reasons for experiencing greater feelings of loneliness is discrimination and stigma, whether it is based on sexual orientation, age, or a combination of the two.

“Yes, because of their sexual orientation, of course, of course. And for being older, I think there is… I don’t know how to say it, it’s a triple discrimination. Because we are older. Because we are older and because they see you as more defenseless, I think that many times LGBT-phobia has a component of cowardice. And receiving LGBT-phobia or an LGBT-phobic aggression and, in this case, against older people, I see it as an act of cowardice, because they pick on people who, perhaps, are not going to be able to defend themselves”.

(I19)

#### 5.3.1. Loneliness Due to Age Discrimination or Ageism

Ageism refers to the stereotyping and discrimination against individuals or groups based on their age, particularly targeting older adults. Many interviewees reported experiencing such age-based discrimination and victimization firsthand.

Interestingly, ageism is also present within the LGBT community itself. As one participant explained as follows:

“Ageism exists within the LGBT environment because we live in a society where diversity is relatively recent. Society is not accustomed to seeing LGBT people age, and the LGBT community is highly ageist. You are pushed out as soon as your physical appearance no longer fits the expected standards of youth, grooming, or whatever else. Once you pass a certain age and no longer meet the physical and aesthetic norms, it’s as if the system rejects you. Young people don’t want to see older people because it reminds them that their own stage of beauty and vitality is temporary”.

(I1)

Another participant highlighted the broader societal preference for youth over experience:

“This society values youth far more than those with more life experience, who have lived through what others are just beginning to face. We want to be recognized and supported; as a group, we do not want to be ignored”.

(I5)

For some, aging brings a desire for authenticity and acceptance:

“At my age, I just want to feel alive, to be comfortable with people as I am, without having to justify anything, and to speak freely”.

(I8)

#### 5.3.2. Loneliness Due to Discrimination for Being LGBT

Discrimination, stigmatization, and harassment based on sexual orientation often lead LGBT individuals to choose loneliness or social isolation as a form of self-protection—that is, the belief that “if I don’t disclose my identity, I won’t be discriminated against”. Additionally, the internalized contempt or negative self-perception caused by bullying can hinder the formation of meaningful relationships, thereby intensifying feelings of loneliness and isolation.

One participant described the profound impact of bullying on their life:

“Bullying, I think, has been the most traumatic experience of my entire life, and during that time, I was completely alone; I was bullied a lot. They did ‘everything’ to me”.

(I16)

Another shared early experiences of exclusion and fear:

“When I was little, around 6 or 7 years old, I liked to wear my hair long. The other kids made fun of me and called me a sissy and other names. Later, when my preferences began to manifest, I tried to go unnoticed. It left a mark on me, and I always tried to be very discreet. In high school, I kept it completely hidden. There was a bit more freedom then, but I was absolutely sure my classmates wouldn’t understand and would harass me again”.

(I17)

#### 5.3.3. Discrimination Within the LGBT Community

However, discrimination can also occur within the LGBT community itself, which is a diverse and plural collective:

“This tendency to segregate and divide us according to the letters (L, G, B, T…) is, in my opinion, a mistake, because what we are really seeking is social diversity”.

(I16)

Grouping people into subcategories can lead to the closure of these groups, connecting individuals with similar desires or intentions, but ultimately isolating them from others. This is a concern because it impoverishes human relationships:

“By joining groups or labeling people, what happens is that the group becomes closed off. It brings together people with shared interests or intentions, but in the end, it’s a closed group disconnected from others. This worries me because it greatly diminishes the richness of human relationships”.

(I8)

Discrimination can also come from within the community itself:

“I have been discriminated against within the group as well. Sometimes it still happens. And of course, I have also faced discrimination from ‘hetero’ people—they have insulted me and so on. But with them, I’ve been able to move past it without much problem”.

(I20)

### 5.4. Loneliness Due to Stigma, Lack of Social Acceptance and/or Self-Acceptance

Stigma and labeling are closely tied to the language and terms used to describe someone. A person can be disqualified simply for belonging to a marginalized group, through the use of language that is rarely neutral, words like faggot, inverted, mentally ill, different, weirdo, feathered, weaponized, or hidden, all of which are deeply hurtful. Such stigmatizing language makes social integration much more difficult for individuals with visibly marked identities. Beyond social discrimination, there is also the weight of societal norms, which in Spain have long been governed by a rigid moral framework:

“Coming to terms with our homosexuality or sexual orientation has led me to rethink the concepts of freedom and responsibility, especially responsibility. We come from a time when freedom was far more limited; it was a very political era”.

(I5)

One interviewee recalled the intense loneliness experienced at a young age as follows: “I felt terrible loneliness when I was 15 because I lived in a socially and economically excluded neighborhood. At that time, you had to be lucky to find someone you could confide in, because otherwise you risked being reported” (I3).

A stigmatized person often sees themselves as equal to others and deserving of the same rights, but their self-concept can be deeply affected by how others define them. This contradiction creates an internal conflict that requires significant effort to resolve.

“When I was 20, I went to a psychiatrist again because I was a mess inside. Outwardly, I was a good son, a model student with excellent grades, and an athlete—everything seemed fine. But inside, I was struggling, because while others saw me as a success, I—especially given the era’s prevailing Catholic values—saw myself as broken”.

(I4)

#### 5.4.1. Recognize and Name It

Stigma, social pressure and the norms that inform social standards are very strong and difficult to change. Participants reported taking various amounts of time to accept their sexual identity, to know themselves, and to recognize themselves. This process of constructing a homosexual identity encompasses different stages throughout the life cycle.

- Sexual Identity Acceptance: A Long and Uneven Journey

The process of accepting one’s sexual identity among older LGBT people is often lengthy and non-linear. Many interviewees shared that it took them decades to fully come to terms with their homosexuality, largely due to the repressive social and moral contexts in which they were raised. This delayed self-acceptance is frequently marked by feelings of isolation, confusion, and internal conflict, such as: “I am 60 years old, and I came out of the closet 3 years ago. I’ve always been a freak. I’ve been alone and isolated in this matter” (I8).

This quotation illustrates how the lack of social support and positive LGBT role models can cause individuals to hide or suppress their identity for most of their lives, resulting in prolonged emotional isolation.

Role of Professional Support and the Power of Language

Some participants found meaningful support through mental health professionals. In some cases, group therapy or simple but empathetic words provided a turning point for self-understanding and self-acceptance: “That psychiatrist did help me, especially because she put me in some groups like group therapy with young people and I saw one who was paranoid, the other one who didn’t know what… And I said: ‘Well, there’s nothing wrong with me! Anyway, I’m just a fag’” (I4).

Through ironic self-reflection, this person expresses a realization that their suffering was more about social rejection than any inherent personal issue. The group environment helped normalize their experience and alleviate their internalized shame.

Another interviewee recalled a deeply affirming experience with a family doctor:

“I was around 65–68. He asked me: ‘What’s wrong?’ I said, ‘Look, I’m an inverted person’. The doctor looked at me, and so did the nurse, and I saw in their faces the maternal and paternal support of a 15-year-old boy asking for help. She came up to me and said: ‘Never again in your life say you’re inverted. There is no such thing. The problem is that they don’t let you be yourself.’ I was amazed. He told me: ‘I can’t help you much, but don’t forget who you are and you must accept and fight for it, even if it’s silently.’ That was enough for me. I just needed a light, someone to tell me something”.

(I3)

This emotionally powerful account shows how even a brief moment of genuine human connection can act as a lifeline for someone struggling with deep internalized stigma. The weight of language—especially terms like “inverted”—also reveals the impact of outdated and damaging societal norms.

- Aging and Identity: A Second Layer of Acceptance

After coming to terms with their sexual identity, many interviewees emphasized the challenge of accepting themselves as aging individuals within a youth-focused LGBT culture. Loss of sexual desirability, reduced social opportunities, and progressive isolation were common themes: “Now as an old man … the body no longer responds. Hunting, flirting … they can’t be the same” (I3).

Here, the participant reflects on the loss of physical vitality and its emotional impact. In LGBT spaces where youth and physical appearance are often prized, aging may feel like social exile: “Making friends is harder for me as I get older. And sex too” (I6).

This quotation signals how aging can impair not just physical intimacy but one’s capacity to create new social bonds, compounding feelings of loneliness: “It’s different to be LGBT at 55 than to be LGBT at 80” (I1).

This simple but profound statement points to a generational gap within the LGBT community itself, where the experiences, values, and visibility of older members often remain unacknowledged: “You realize that old age lurks… I’m withdrawing. As the years go by, my partner and I are closing the circle, one with the other, alone. I don’t want to enter the ‘old man’ model” (I9).

This participant describes a process of voluntary withdrawal, driven by the desire to maintain autonomy and dignity. Yet it also reflects a form of self-isolation linked to the fear of being reduced to a stereotype of aging and dependence.

#### 5.4.2. Loneliness Due to Lack of Acceptance of Sexual Orientation

- Towards Identity Acceptance

The path from non-acceptance to full recognition of one’s sexual identity is often complex and prolonged for many LGBT individuals. This journey typically involves navigating both external social pressures and internal psychological struggles. It is not simply a personal journey, but a socially conditioned process shaped by discrimination, homophobia, stereotypes, persecution, and—until relatively recently in Spain—criminalization. These societal factors often lead to personal experiences of victimization, denial, secrecy, and internalized shame.

This short yet powerful statement reveals a common strategy among older generations of LGBT individuals: conforming to heteronormative expectations as a means of survival or social acceptance.

“I came back to Spain still, more or less, in the closet… I had had a partner, a girl, of course, and since I had a girlfriend on top of that, I was even more framed because I covered the social expectations and I was not fully accepted”.

(I4)

Marrying and having children became a way to fit into societal norms, even if it meant denying one’s true identity: “I got married and had a daughter” (I14).

The participant describe how maintaining a heterosexual appearance helped to mask their sexual orientation. This ‘double life’ highlights the internal conflict between authentic identity and social acceptance, and the exhausting nature of living behind a façade.

- Consequences of Non-Acceptance

The effects of societal rejection often manifest as deep personal insecurity and prolonged experiences of loneliness, especially during periods of identity denial or concealment.

“I remember that I had to face my own fears of loneliness, to say: ‘Well, why do you panic about loneliness?’ Right? And it was probably because of a history of feeling undervalued myself, of insecurities, of… well, of a family that I came from, who were not at all loving”.

(I5)

This participant explains how early familial experiences of emotional neglect, compounded by societal rejection, can shape one’s self-perception and fears of abandonment. For many LGBT individuals, loneliness stems not only from social isolation but from internalized feelings of unworthiness: “Regarding loneliness, I could try to synthesize a first phase of a lot of loneliness while I did not accept myself, especially in identity, sexuality” (I4).

This quotation reflects the deep connection between self-acceptance and emotional well-being. For this person, loneliness was not just circumstantial but tied directly to a period of identity confusion and internal denial. It suggests that the journey to self-acceptance can be accompanied by a painful sense of disconnection, from others, and from oneself.

#### 5.4.3. Fear of Rejection and Social Homophobia. Struggling to “Come out of the Closet” and Express Themselves Openly

- Fear of Rejection, Isolation, and the Costs of Self-Protection

For many LGBT individuals, particularly older adults, the fear of rejection often results in voluntary isolation. Avoiding disclosure and distancing oneself from others become forms of self-protection against discrimination. As noted by Perone et al. (2020), this long-term marginalization leads to heightened social isolation in later life, which further contributes to vulnerability to loneliness: “Loneliness is something that is part of us. And then, well, I have to tell you that I didn’t come out of the closet until I was 34” (I1).

This testimony reflects how internalized stigma and social fear can delay the process of coming out, embedding loneliness as a persistent life companion: “Well, my father was very homophobic… and at work there were very motherfuckers, that’s for sure. One of my best friends said that if she had a homosexual son, she would throw him out of the house. It made me feel…” (I11).

This quotation illustrates how family rejection and hostile work environments can discourage individuals from revealing their sexual orientation, reinforcing isolation and fear: “As special moments, at the age of 18 I remember that I went to the telephone of hope and to a psychiatrist. He wanted to give me hormones… He said I had to affirm my masculinity… and then I stopped going to the psychiatrist” (I4).

This experience demonstrates the damaging impact of pathologizing homosexuality, especially during a vulnerable stage of identity formation. Rather than receiving support, this participant was offered treatment to “correct” his identity, deepening his isolation.

- Coming Out as a Critical but Painful Milestone

Coming out is a pivotal life event that is often accompanied by fear, emotional distress, and the loss of social ties. While it can be liberating, it frequently involves negative reactions from others and the need to justify one’s identity: “There were friendships that I still maintain and other friendships that have fallen by the wayside, because they asked me for justifications and I said: ‘Well, why do I have to justify myself? You justify why you are heterosexual!’” (I16).

Here, the participant questions the asymmetrical social expectations placed on LGBT individuals to explain themselves, highlighting the unfair burden of justification.

“There is also the problem that, when it comes to opening up, if the other person is heterosexual, you don’t know whether to open up or not… I think: ‘What position will this person have on homosexuality?’ Even if it’s a bit strong, I think about it. Because I say: maybe I open up, and it hurts me”.

(I6)

This quotation highlights the constant self-monitoring and risk assessment that LGBT individuals perform when navigating social interactions, particularly in environments perceived as potentially unwelcoming or unsafe, reinforcing how fear of rejection shapes cautious behavior: “Some LGBT-phobic attitudes are internalized… there is always a reluctance to show themselves as they are…” (I19).

This insight speaks to internalized homophobia, where negative societal views are absorbed by individuals, even after gaining rights or outwardly expressing pride: “But that not all topics can be talked about openly, without any kind of consequences or repercussions” (I8).

Despite progress, this participant underscores how social censorship still exists, and full freedom of expression remains elusive for many LGBT individuals

- Geographic Relocation (sexual exile) as a Search for Freedom

Some participants described geographical relocation or sexual exile, forced migration because of their LGBTQ+ identity, leaving their hometowns or even countries as a strategy to escape stigmatizing environments and to gain the freedom to live authentically. However, this often entails starting over and enduring new forms of loneliness: “Those of us who have broken with the past have no life here. I have only been in this city for 3 years, and on top of that, the pandemic arrives” (I6).

Relocation is shown here as a double-edged sword: a path to authenticity, but also one that severs ties with one’s previous support systems, especially painful in times of crisis.

“At the age of 30 I left my friends and came here. Between 30 and 40 years old I made my transition. Since then, I am in the Lambda Association… I felt free”.

(I12)

By contrast, this person associates relocation with empowerment and community building, particularly after joining an LGBT organization.

“In 1985, I went to another country, to start another life… I no longer had the burden of responsibility… I was starting to come out but the feeling of loneliness was very great and it was also a real loneliness because it was starting a new life in a place where I did not know anyone”.

(I4)

This reflection captures the emotional paradox of relocation: even in freedom, the loss of familiarity and existing relationships can deepen emotional isolation.

#### 5.4.4. Homosexuality and Living with HIV: “Coming out of Two Closets”

The social rejection of HIV-positive individuals remains deeply rooted—even within the LGBT community. Although antiretroviral therapy (such as AZT) has been available for over 30 years and has significantly reduced AIDS-related deaths, medical advances have not been enough to dismantle the stigma and fear surrounding an HIV diagnosis. These testimonies reflect the emotional, social, and psychological burden still faced by those living with the virus.

“I had to come out of two closets. One for being homosexual, well, okay, fine, whatever you want to call it, I don’t care, and one for being HIV-positive. I never really came out of the closet because I’m gay, I didn’t have a closet. But I had another one, and it was hard to come out of that one, and it still is”.

(I13)

This “second closet” of seropositivity often remains invisible and unspoken. For many, the fear of rejection or judgment continues to shape their relationships and self-perception:

“I’ve been very afraid of relationships. Being HIV-positive, I haven’t had the courage—let’s put it that way—to say: ‘I am HIV-positive’. Everyone seems so relaxed about it today, but 30 years ago, it wasn’t like that. So of course, that fear is still there, even if just a little. It taught me I had to fend for myself. Loneliness has become a sort of travel companion. I wouldn’t say I’m comfortable with it, but I don’t dislike it either”.

(I13)

Even within social circles that should offer support, silence and stigma can prevail. One participant recalled the advice he received from a friend: “A friend told me: ‘When they ask you if you’re HIV-positive, just say you’re getting tested or something. Don’t say you are, because you screw up the whole group’” (I3).

The weight of such internalized stigma can leave a lasting impact on one’s identity and sense of belonging: “Even today, I live with being HIV-positive as if it were a curse” (I3).

These narratives underscore a persistent tension between medical progress and social acceptance. While treatment has transformed HIV into a manageable chronic condition, the emotional and social landscapes have not evolved at the same pace. The voices of those living with HIV remind us that stigma, especially when coming from within one’s own community, can be as harmful as the virus itself. Addressing this requires not only public health solutions, but a cultural shift toward empathy, visibility, and solidarity.

### 5.5. Loneliness Due to Absences

#### 5.5.1. The Absence of Socialization Spaces

The lack of community spaces where one can develop social relationships is also perceived as a possible contributor to undesired loneliness.

“There are other people who do not find spaces where they can interact, communicate and so on, because, well, life has not… has not made it easy for them, that is to say, those ways of approaching associations, that and more, and showing themselves as they are. That is to say, we have isolated ourselves a lot in our little hive and sometimes, with age and so on, with television, with your books, with a series of things, you become more and more isolated”.

(I2)

A recurring theme among interviewees was the need for physical and social spaces where LGBT individuals—especially older adults—can gather, connect, and interact safely and openly. These are not just leisure spaces, but symbolic and practical environments where people can feel visible, supported, and equal. The participants highlighted both the importance of LGBT-specific spaces and the need for intergenerational and community-based spaces within neighborhoods. One interviewee simply expressed “That there were places to be in the neighborhood, that I could be with people more or less similar, the same” (I2), to emphasize how fundamental this need is.

This quotation, though concise, speaks volumes about the longing for a sense of belonging and everyday normalcy within one’s own community. The emphasis is not only on LGBT identity but also on neighborhood proximity and integration: “It is good that spaces are created, like the one offered by Senior Group of Lambda Association and others…, but also in the neighborhoods there are associations and collective spaces to try to create groups where people interact; LGBT people and others, because they have their places and meeting places” (I2).

Here, the interviewee recognizes the value of targeted initiatives (such as the Lambda Association’s Senior Group), but also emphasizes the importance of integration: LGBT people should have access to broader community life without being confined to exclusive spaces.

“I would love to find an area where people, of our age or younger, could have our spaces, meetings and rooms where we could interact. The districts of this city have not advanced in spaces where people can get to know each other, and show themselves as they are, without having to hide anything”.

(I2)

The emotional depth in these quotations reflects a strong desire to live openly, which underscores the importance of creating inclusive, non-judgmental spaces.

The testimonies reveal a pressing need for inclusive, intergenerational, and local spaces where LGBT individuals—especially older adults—can build community. While LGBT-specific associations offer crucial support, many participants long for everyday visibility and belonging within their own neighborhoods. The absence of such spaces not only limits opportunities for social interaction but reinforces feelings of isolation and marginalization. Addressing this gap requires urban and social planning that centers visibility, inclusion, and intergenerational dialogue, ensuring that LGBT people can age with dignity, connection, and pride.

#### 5.5.2. The Generation Gap

The intergenerational gap, that is, the differences and separation between younger and older generations, can also trigger feelings of loneliness.

“And within the LGBT collective, we all know that older people are a group where young people have always prevailed a lot, because of fashion, because of the culture and so on. And older people have been more cornered, pushed aside, also culturally because we come from another era where at the beginning there were more people in the closet, so it has been more difficult to elbow our way in, even within our collective, when we are also getting older”.

(I2)

However, this segmentation is often produced by the organizations themselves.

“I was talking about segmentation, not only because of age in the collective in general, but also the LGBT organizations themselves, like what is happening in our organization, which creates differentiated groups that do not allow interaction between different generations”.

(I1)

#### 5.5.3. Loneliness Due to Digital Divide in a Complex and Technologized World

Another factor that can motivate the appearance of loneliness is the “digital divide”, inequality related to the accessibility, use, or impact of information and communication technologies between social groups due to economic, age, or cultural factors. Actions as common as sending an e-mail can be an obstacle to social participation and the maintenance or creation of social ties.

“What is causing the most loneliness with this pandemic is the digital divide, that is, the isolation that is taking place, especially among older people, whether LGBT or not. Because there are people who maybe we do know how to communicate and so on, but there are other people who have this impossibility because they do not know, do not control everything very well, all these new forms of meetings… To say that of a group of about 25 people, there are 5 of us, the ones who are moving a little in the chat”.

(I1)

Participants also reflected on the disconnect brought about by the emergence of new digital forms of social interaction:

“It is as if we were talking or starting from completely different situations, probably we do continue with the concept and the canon of looking for relationships with people face to face, of talking more, while young people are much more digitalized, they do not need that kind of things so much, that is an important gap”.

(I5)

## 6. Loneliness Due to the COVID-19 Pandemic

Another noteworthy revelation resulting from this study was how loneliness became more visible during the COVID-19 pandemic. Although not all people experienced this in the same way, it has been a great handicap for some, often because their needs were forgotten and became invisible.

“We used to meet on Fridays and it was a way to interact, to go to dinner, to talk and tell each other about our experiences, but in these times of pandemic, we are limited, but above all what limits us is the digital divide. It is a pity that sometimes, because of the digital divide, a number of people are not there”.

(I2)

Some participants expressed that the confinement and restrictive measures increased the feelings of loneliness that had already existed previously.

“I live alone, except that I have two cats that keep me company, and with little social contact now that they have restricted us a lot. At least, I have work and interact with people at work and I go for a walk in the afternoons; sometimes, I meet some friends in the neighborhood. In general, I cope well with loneliness, because it is a chosen loneliness, but with the pandemic I do not cope so well, because that part of socialization that I had with my friends, going out, meeting up, getting together… well, no more”.

(I11)

Participants not only highlighted the gap introduced by emerging digital forms of interaction but underscored the persistent need for opportunities to socialize.

“I prefer to live alone. Thing is that with the pandemic it’s… It’s been a bit bad. Quite bad. I need to go out, I need to see people, I need this social life to make up for the moments of that chosen solitude, that socialization and so on”.

(I11)

## 7. Ways to Cope with Loneliness

The coping strategies most commonly used in older people are active coping (acceptance, actively searching for people and spaces for socialization, etc.), self-distraction, positive reinterpretation, and religion. Active forms of coping strategies are the efforts made to cope with the situation, and these have positive effects on adaptation; on the contrary, passive (or avoidant) forms correspond to avoidance and denial behaviors, and are not very useful for adaptation.

When talking about unwanted loneliness, participants discussed past and present aspects of their lives that have made them more resilient and named different strategies they use to cope with various challenges. These ranged from more individual coping methods, focused on their own strengths, to those related to social or family support.

“The thing about loneliness is that it’s okay to accept that, at any time, for any circumstance, we can be alone in life. I don’t know, I have always felt that way, I think that also depends a lot on whether a person has worked at it. And we have not been taught that, we have not been taught to have our spaces, our moments of solitude or of sharing”.

(I19)

### 7.1. Relationships with Friends, Family, and Partners

Most of the people interviewed agreed that bonding with friends, partners, and family is one of the most important elements to reduce unwanted loneliness: “I take good care of my relationships with friends, I try to take good care of my family and my partner, so for me that is the most important thing there is” (I12). This statement echoes the same emphasis on the need for social connection: “My way of solving loneliness is to take good care of relationships, which, for me, the most important thing is the bond with people, for me it is fundamental” (I11).

As key factors in the prevention of loneliness in the participants, the role of social participation and the support of the peer group is highlighted as follows: “When I want to escape from loneliness, I go to my friends and escape from loneliness” (I13).

Friendships often become “chosen families”, providing necessary support in difficult life situations.

“I think that loneliness also has to do with the ability to… to feel accompanied… I think it is when a person is able to connect with others, with friends or with the person next to him, to have empathy with the person you are with. For me, I think that’s the most important thing to not feel alone. If you are only looking out for your needs, then since they don’t do what you want, you feel lonely because they don’t respond to you”.

(I12)

On the other hand, collective actions help to increase one’s sense of belonging to the group, and LGBT people can see themselves integrated into joint actions and feel useful, which, in addition, helps to improve their psychological well-being and social integration.

“Now, at this time, as I am older, I think I have more resources, more experience, and I have some pillars on which my life is based to feel accompanied and feel good. First of all, my husband, I have been married for 5 years, but I have been with him for 18 years. For me, the most important thing is to be with him. Friends are a very important part for me to feel good. And family too; I get along well with my sister and my nieces and nephews and I try to feel accompanied. For me it is very important because as I have felt very lonely many times and a lot of time for me feeling accompanied makes me feel safe, feel good”.

(I12)

### 7.2. Social Participation

In general, the interviewees stated that having a good social network prevents the appearance of unwanted loneliness, and that participation in the LGBT Seniors Group has been of great help to them. For this reason, the relations of the LGBT person with the organizations of the collective to which they belong are decisive. However, in order to be part of such a collective, the person must socially assume the stigma, questioning it while, at the same time, claiming it; in addition, they must have previously accepted their social identity, making it part of their personal identity: “When you feel well surrounded and participate it is very difficult to feel alone” (I12).

Interactions of a binding type, typical of quite homogeneous groups, as may be the case for LGBT associations, and of groups of older LGBT people in particular, tend to be stable and develop solidarity and specific reciprocities, since they are configured as collaborative networks based on identity.

“To reduce loneliness, obviously one has to associate with people more or less, who have some emotional ties or who have some ideological link or in this case would be egalitarian in the issue of sexuality. I think we have to learn to coexist much better, even heterosexual people can, must, learn to coexist also much more with the needs that we have as a whole LGBT collective”.

(I16)

Several interviewees underlined how meaningful the support from the group had been:

“For me, being in the Senior Group of Lambda Association is like a very big relief in which you have meetings, you can meet people, you can socialize in a different way. You have a need to meet people. You are at a stage in your life that is practically limiting in some aspects, so you do feel very conditioned. The group helped me a lot”.

(I8)

### 7.3. Acceptance and Adaptability

One of the main coping strategies that helped the participants accept loneliness in a more constructive way was their ability to adapt to change and focus on the present. Rather than projecting fears or expectations onto an uncertain future, many interviewees described a conscious effort to live day by day, embracing unpredictability, accepting solitude, and finding contentment in the everyday. This flexible mindset was repeatedly identified as a key mechanism for emotional resilience and psychological balance.

One participant described this process in striking terms, emphasizing both acceptance and adaptability:

“But it is true that I learned not to wait. What is coming to me today? This is what’s coming today, that’s what’s coming today, that’s what’s coming today. That I have my old age here? Well, I have my old age there (laughs). As far as I can, I will try to live it as well as possible. I will try to enjoy it in the best possible way. My life has been like that—I have been adapting to situations: having a job, not having a job, if I explain it, I don’t explain it… I have adapted to being alone. If someone comes, welcome; if they don’t, they don’t need to. So, it’s living little by little; adapting to whatever comes my way”.

(I13)

This testimony reflects a deep internalization of adaptive coping, where the individual does not deny adversity but chooses to flow with it. The notion of “living little by little” captures a philosophy rooted in present awareness and a resistance to anxiety-inducing projections about the future.

The same participant summarized this approach in a more concise but equally powerful way: “I live day by day and nothing else. My life has been a continuous adaptation” (I13).

Here, temporal focus and emotional flexibility become intertwined. By anchoring themselves in the present and normalizing change as a constant, individuals not only mitigate the emotional weight of loneliness but reclaim a sense of agency.

In sum, these narratives suggest that adaptive acceptance rooted in present-focused living functions as a crucial resilience strategy. For individuals facing prolonged solitude or instability, this mindset fosters emotional endurance, reduces existential anxiety, and affirms the possibility of well-being even in contexts of loss or disconnection.

### 7.4. Entertainment and Leisure

When discussing strategies for coping with loneliness and psychosocial distress, many interviewees emphasized the role of distraction as a coping strategy, particularly through cultural and recreational activities. These include engaging with music, reading, cinema, theater, urban exploration, travel, and the use of digital technologies. Such activities not only provide emotional relief but foster a sense of purpose and connection in the absence of close interpersonal relationships.

One participant described the Internet as a space of immersion and discovery, offering both entertainment and unexpected social encounters:

“The world of the Internet is a world in which I immerse myself at all levels without any problem. It is something I am passionate about, and there I have discovered surprising contacts that I did not know about. Now I see that the Internet is a whole world, and that entertains me and helps me cope”.

(I14)

This testimony reflects how digital environments can serve as a form of social compensation, especially for those facing limitations in physical or local interaction. Online engagement becomes more than mere distraction; it is a form of agency in navigating solitude.

Other interviewees highlighted the therapeutic role of cultural curiosity and physical movement. One participant explained: “I have felt lonely, but since I have always been very curious, I have made up for the loneliness of being alone, of not knowing anyone, by traveling wherever I can, going around the city, going to the theater, to the movies. All of that has helped me a lot.” (I4)

Here, mobility and cultural consumption are framed as active strategies to counter isolation. Rather than passively enduring loneliness, the participant seeks stimulation and connection through art, public spaces, and discovery.

Another respondent articulated this coping mechanism in simpler, yet deeply telling terms as follows: “I always fill my life with activities … to avoid thinking” (I9).

This statement suggests a more defensive or avoidant form of distraction, underscoring how busyness can act as an emotional shield. The deliberate occupation of time serves to displace intrusive or distressing thoughts, highlighting the dual function of distraction: both as self-care and as avoidance.

Overall, these narratives illustrate that distraction is not merely a way to pass time, but rather a complex psychosocial strategy. Whether through digital exploration, cultural participation, or physical activity, it allows individuals to reshape their experience of loneliness, reinforcing autonomy and emotional resilience in the face of social fragmentation.

## 8. Discussion

The purpose of this study was to learn about homosexual men’s feelings of loneliness and their relationship with experiences of discrimination throughout their life, and to analyze the impact of their experiences of discrimination and stigma on the onset of loneliness. Our analysis identified the main triggers of this loneliness, highlighting the importance of loss, deprivation, and absence, as well as their impact on mental health and well-being, as demonstrated by other researchers (Nowaskie & Roesler, 2022; Firk et al., 2023; Bavinton et al., 2022; Akré et al., 2021). A clear link between discrimination, stigma, and loneliness was also revealed.

### 8.1. Loneliness

Firstly, this research revealed the various situations of loneliness that adult homosexual men have experienced throughout their lives and continue to experience today. The qualitative analyses allowed us to find commonalities and individual nuances in the personal experiences of each participant.

Authors such as Ribeiro-Gonçalves et al. (2019), in their research on social connectedness, showed that LGBT people report greater loneliness, less social support, and less face-to-face contact than heterosexual people. Thus, individuals identifying as LGBT were less socially connected than people who did not identify as LGBT. The differences in social isolation and loneliness between these groups may be partially attributed to sociodemographic differences, as LGBT people are more likely to be childless and/or live alone. Our findings show similar results, with one in every two participants living alone (50%), and for only one of the participants having a child.

LGBT adults are more likely to live alone, not be in a relationship, and not have children (Fredriksen-Goldsen, 2011). They tend to have less contact with other people, which gives them fewer opportunities to form and maintain relationships throughout their lives.

According to the social convoy model (Antonucci et al., 2014), social relationships consist of multiple distinct elements, including structure, function, and quality. LGBT adults may rely more heavily on their social networks—especially meaningful friendships—for their mental well-being (Tester & Wright, 2017). An empirical study by Fredriksen-Goldsen et al. (2013) found similar results: greater social support and larger social network size served as protective factors against negative health outcomes in older LGBT adults.

### 8.2. Discrimination

Secondly, research in the *minority stress theory* states that sexual minorities face many stressful psychosocial events and stigmatizing conditions at the structural level in a heteronormative society, and indicates that homophobic and ageist attitudes in society result in double discrimination, or triple discrimination in the case of people living with HIV, who may suffer greater discrimination and stigma from society, from their peers, and even self-stigma, which can lead to greater unwanted loneliness and isolation, and in some cases may even lead to mental health problems. These experiences have a damaging effect on a person’s identity.

It has also been shown that people’s needs differ with age, as they experience different changes, different health problems, face more barriers, and become less visible. Thus, loneliness manifests to a greater extent in older people due to risk factors such as the appearance of health problems or reduced social participation.

This work also describes the different coping strategies the interviewees used to cope with loneliness, such as resilience, the ability to adapt to changes, social support, leisure, acceptance, and spaces for collective meetings and gatherings, including participation in LGBT associations.

### 8.3. COVID-19 Impact

Analyses have shown that loneliness, due to social confinement and distancing, is associated with depressive symptomatology in this population group. Therefore, policy makers and social and health care professionals should consider the importance of social interactions in all people, especially in stigmatized groups, as stated by Ribeiro-Gonçalves et al. (2019). Gato et al. (2021) explored how the psychosocial effects of the pandemic affected the mental health of LGBT young adults from six countries (Portugal, UK, Italy, Brazil, Chile, and Sweden) who were confined with their parents during the lockdown period (N = 1934), and found that rates of depression and anxiety were higher among those participants who reported feeling more emotionally affected by the pandemic, those who felt uncomfortable at home, or those who were isolated from non-LGBT friends. In light of this finding, they recommended that LGBT community groups, as well as health and educational services, should be particularly attentive to the needs of LGBT young adults during health crises.

Some studies have presented interesting results, a good example being the study by Firk et al., 2023, who conducted a cross-sectional online survey during the initial COVID-19 lockdown in Germany. This work complements the existing findings and provides a detailed analysis of interviews, making it possible to consider the situation of older gay men in greater depth.

### 8.4. Social Needs and Some Proposals

This work also highlights the importance of support networks that are sensitive to sexual diversity, which can help prevent feelings of insecurity and discomfort, as well as behaviors that negatively affect well-being. To reduce loneliness, it is important to build associations between people within the same group. It is therefore necessary to create networks of LGBT people and foster a sense of community within this demographic. Loneliness is linked to mental health problems and suicidal ideation, and given the alarming number of suicides in recent years, these findings are in need of attention (Eres et al., 2021).

In terms of this study’s strengths, the in-depth interviews and the various focus group sessions allowed us to delve deeper into issues of concern. The participants reflected on issues that they said they do not usually discuss with anyone and that they are particularly concerned about. Despite the participants’ health problems, discrimination, and economic problems, we observed enormous resilience and satisfaction with life. Perhaps having seen so many friends die when they were 20–25 years old (in the 1980s–1990s in Spain, as a result of the HIV epidemic) has inspired this demographic to make the most of every moment of their life and place a high value on their links with their friendship networks.

### 8.5. Limitations

Some limitations of this study are as follows: most of the participants belonged to an LGBT association; additionally, conducting the focus groups and interviews online meant that our study group was composed solely of people with Internet access. We were unable to interview very elderly people (those over 80 years old), whose life situations may differ because they belong to a different cohort.

This study also has some further limitations, such as its cross-sectional design, its qualitative methodology, which does not allow for causal analysis, and the small number of participants, which could affect its generalizability. The fact that the sample includes only homosexual men may also be a cause for concern with regard to gender equality or of extrapolation of the results to the rest of the LGBT collective. Nevertheless, it offers a pioneering view of the different experiences of loneliness in this population group in Spain. Furthermore, this study was conducted during the COVID-19 pandemic, so it provides important information on the specific experiences of adult homosexual men and highlights the need for additional research in this field. The pandemic highlighted the many problems faced by vulnerable people throughout their lives, and these findings have been supported by various authors (Gato et al., 2021).

Another potential limitation is that only gay men were interviewed; however, this was also a strength, as it allowed for a better understanding of particularities that might have been diluted in research involving LGBT people as a whole.

## 9. Conclusions

Older adults who identify as LGBT constitute a growing and increasingly recognized demographic within the aging population. Despite greater social visibility, this group continues to experience pronounced health disparities relative to their heterosexual peers, particularly in the domains of mental health and emotional wellbeing (Fredriksen-Goldsen et al., 2013). A smaller social network is consistently linked to heightened levels of loneliness (Czaja et al., 2021). While social networks comprise various structural dimensions, such as frequency of interaction and physical proximity to network members, the positive relationship between greater network size and improved mental health remains significant, even when these factors are accounted for. This protective role of larger social networks has likewise been observed among older LGBT populations (Erosheva et al., 2016; Kim et al., 2017). A robust body of empirical research underscores the critical role of social relationships in shaping mental health outcomes (Antonucci et al., 2014), while chronic experiences of loneliness have been consistently associated with adverse psychological and physiological consequences. However, few studies have explored the relationships among these three social network characteristics in Spanish older adults as factors that may impact emotional wellbeing.

The results show several problems faced by LGBT people during the aging process.

In particular, the participants mentioned the fear of aging; and on the other hand, they mentioned the thought of an old age in which they need care and have no one to look after them and no suitable places for their care, with professionals who are sufficiently trained in gender diversity.

The timing of the interviews, which took place in the midst of the various waves of COVID-19, allowed us to learn about the impact COVID-19 had on the participants’ lives, and the associated feelings of loneliness. The interviews allowed us to delve into the loneliness and discrimination suffered by the participants, as well as the different methods they used to cope with the loneliness. The specific case of older LGBT people with HIV, who noted loneliness in the face of their health problems, coping with loss, and living each day as if it were their last, is worth studying in depth, and we have not found many articles that refer exclusively to this specific topic.

Higher levels of loneliness among LGBT individuals, compared to heterosexuals, have also been demonstrated in previous studies, and are related to the minority stress model (Fredriksen-Goldsen et al., 2013; Kim & Fredriksen-Goldsen, 2014). Weaker social networks may stem from disconnected biological families and difficulties in finding like-minded people (for example, older LGBT adults). The formation of social networks among LGBT older adults has historically been constrained by experiences of familial rejection and the legal exclusion from marriage and adoption rights. These structural and social limitations have often resulted in reduced access to traditional kinship-based support systems. In response, many LGBT older individuals have cultivated alternative support structures—commonly referred to as “chosen families”—comprised largely of age peers and other members of the LGBT community. These identity-affirming networks have been positively associated with emotional wellbeing (Breder & Bockting, 2023). Moreover, the expansion of such networks may enhance the capacity to address the diverse and evolving social needs of this population.

In line with the previous research, loneliness is associated with marital status, age, gender, mental health, physical health, educational level, and income. Although this study is qualitative and we did not perform correlation analyses, we observed that these variables tend to occur together.

The results have implications for both research and interventions. This study advances the literature by employing a qualitative methodology, enabling a nuanced understanding of the diverse aging experiences among LGBT older adults. The findings reveal that structural dimensions of social networks—such as network size and shared identity—are closely linked to emotional wellbeing, with perceived social support playing a mediating role. These insights can inform the design of public policies and community-based interventions aimed at strengthening social support systems and promoting health equity for LGBT older populations.

Our study is novel and timely in demonstrating the higher prevalence of loneliness in two under-represented groups of older adults with the potential consequences this may have for their health and wellbeing in later life.

## Figures and Tables

**Table 1:** Results for sociodemographic variables.

Participants	Age	Health Status	Work Status	Ways of Living
I1	57	Good	Full-time employee (social worker)	Living as a couple
I2	61	Some problems	Unemployed	Living with mother
I3	68	Good	Retired	Living alone
I4	64	Good	Retired (primary school teacher)	Living alone
I5	60	Some problems	Part-time employee	Living as a couple
I6	44	Mental health problems	Disabled pensioner	Living with mother
I7	55	Blindness	Disabled pensioner	Living as a couple
I8	60	Good	Farmer	Living with mother and sisters
I9	57	Mental health problems, 65% disability	Disabled pensioner	Living as a couple
I10	64	Mental health problems	Unemployed	Living as a couple
I11	54	Cardiovascular problems	Full-time employee (in charge of risk prevention at work)	Living alone
I12	63	Good	Retired (pharmacist)	Living as a couple
I13	53	HIV	Full-time employee (prevention of risks at work)	Living alone
I14	68	Good level	Retired (high school teacher)	Living alone
I15	53	Some problems	Part-time employee	Living alone
I16	60	Some problems	Unemployed	Living as a couple
I17	54	Good	Part-time employee	Living alone
I18	51	Good	Full-time employee	Living alone
I19	45	Good	Full-time employee	Living alone
I20	43	Good	Full-time employee	Living alone

**Table 2:** Coding in categories and subcategories.

1. CHOSEN OR DESIRED SOLITUDE		
2. UNDESIRED LONELINESS	2.1. Loneliness linked to old age and loneliness linked to being LGBT	
	2.2. Loneliness due to losses and shortages	2.2.1. Loss of partner or fear of damaging relationships
		2.2.2. HIV losses
		2.2.3. Lack of family support
	2.3. Loneliness due to discrimination	2.3.1. Loneliness due to ageism: age discrimination
		2.3.2. Loneliness due to discrimination for being LGBT
		2.3.3. Discrimination within the LGBT community
	2.4. Loneliness due to stigma, lack of social acceptance and/or self-acceptance	2.4.1. Recognize and name it
		2.4.2. Loneliness due to lack of acceptance of sexual orientation
		2.4.3. Fear of rejection and social homophobia. Struggle to “come out of the closet” and express themselves openly.
		2.4.4. Homosexuality and HIV: “Coming out of two closets”.
	2.5. Loneliness due to absences	2.5.1. Loneliness due to the absence of socialization spaces
		2.5.2. Loneliness due to a generation gap
		2.5.3. Loneliness due to digital divide in a complex and technologized world
3. LONELINESS DUE TO THE COVID-19 PANDEMIC		
4. WAYS TO COPE WITH LONELINESS	4.1. Relationships with friends, family, and partners	
	4.2. Social participation	
	4.3. Acceptance and adaptability	
	4.4. Entertainment and leisure	

## Data Availability

The data collected in the research (recordings of the interviews and transcripts in Spanish) are available to other researchers upon request.
